# Low fT3 is associated with diminished health-related quality of life in patients with acute coronary syndrome treated with drug-eluting stent: a longitudinal observational study

**DOI:** 10.18632/oncotarget.21811

**Published:** 2017-10-10

**Authors:** Chao Xue, Ling Bian, Yu Shui Xie, Zhao Fang Yin, Zuo Jun Xu, Qi Zhi Chen, Hui Li Zhang, Yu Qi Fan, Run Du, Chang Qian Wang

**Affiliations:** ^1^ Department of Cardiology, Ninth People’s Hospital, Shanghai Jiao Tong University School of Medicine, Shanghai 200011, People’s Republic of China; ^2^ Department of Cardiology, Ruijin Hospital, Shanghai Jiao Tong University School of Medicine, Shanghai 200025, People’s Republic of China

**Keywords:** health-related quality of life, free triiodothyronine, drug-eluting stent, acute coronary syndrome

## Abstract

Acute coronary syndrome (ACS) patients with low triiodothyronine (T3) syndrome characterized by low free T3 (fT3) levels with normal thyroxine (T4) and thyroid-stimulating hormone (TSH) have a higher rate of death. The impact of fT3 on Health related quality of life (HRQOL) in patients with ACS is still unknown. 528 ACS patients treated with drug-eluting stent (DES) were included in this prospective, observational study. Patients were classified into low fT3 group (n=126) and normal fT3 group (n=402) according to serum fT3 level. Every patient was prospectively interviewed at baseline and 1 year following percutaneous coronary intervention (PCI). HRQOL was assessed with the use of the Medical Outcomes Study 36-Item Short-Form Health Survey (SF-36). Low fT3 patients had poorer HRQOL than normal fT3 patients both at baseline and 1-year follow-up (all p<0.05). During 1-year follow-up, HRQOL scores for all patients were significantly higher than baseline. Low fT3 patients had lesser gains in physical functioning, bodily pain, general health, vitality, social functioning and role emotional (all p<0.05). Generally, low fT3 patients demonstrated less improvement in Physical Component Score (PCS) (p=0.008) and Mental Component Score (MCS) (p=0.001). The percentage of patients reaching MCID for PCS and MCS was lower in low fT3 group than that in normal fT3 group (p<0.001). Multivariate linear regression analyses showed that low level of fT3 was an independent risk factor for PCS and MCS improvements. In conclusion, a low fT3 level is a predictor of worse HRQOL improvement in ACS patients treated with DES.

## INTRODUCTION

Acute coronary syndrome (ACS) refers to a spectrum of life-threatening cardiac diseases in the world including China, and this disease not only increases the mortality but also impairs the health-related quality of life (HRQOL) [1]. HRQOL is increasing recognized as an important endpoint in ACS patients as a result of the reduced mortality. Poor HRQOL has been shown to predict adverse clinical outcomes [2, 3]. Modern treatments of ACS focus not only on improving life expectancy, but also on improving HRQOL in the clinical decision-making process and in the determination of therapeutic benefit. Given the importance of HRQOL, there is a need to identify which characteristics are predictors of subsequent impairments or improvements in HRQOL.

Thyroid hormone plays an important role in the cardiovascular system [4]. Studies have shown that thyroid hormone metabolism changed after acute myocardial infarction or cardiac surgery, resulting in low serum triiodothyronine (T3) levels despite normal thyroid-stimulating hormone (TSH) and thyroxine (T4) [5-8]. Accumulating studies have found that the thyroid hormone level, especially free T3 (fT3), has emerged as a strong prognostic determinant in chronic heart failure [9, 10] and acute myocardial infarction [11]. ACS patients with low level of fT3 have a poorer prognosis [12]. However, the relationship between fT3 and HRQOL in ACS patients has not been comprehensively studied. The purpose of this study is to evaluate the influence of fT3 on long-term HRQOL in ACS patients treated with drug-eluting stents (DES).

## RESULTS

### Patient population

A total of 613 consecutive patients were enrolled and completed the baseline HRQOL instrument. 528 (86.1%) patients finally completed the 1-year quality of life assessment and were enrolled into our study for subsequent analysis. Of them, 402 (76.1%) patients had fT3 within the normal range, 126 (23.9%) patients had suppressed fT3 values. Fourteen patients died before 1-year interview. There were no significant differences between non-respondents and respondents in terms of gender and most baseline characteristics except that non-respondents were more likely to be younger (62.04 years vs. 65.84 years, p=0.015).

### Baseline clinical characteristics

The clinical characteristics, angiographic features and laboratory parameters of the patients are shown in Table 1. Compared to normal fT3 patients, low fT3 patients were more often female (35.7% vs. 24.6%, p=0.023), and less likely to have a history of prior percutaneous coronary intervention (PCI) (23.8% vs. 39.5%, p=0.001). Meanwhile, they were more likely to have a history of diabetes (37.3% vs. 30.1%, p=0.043), hypercholesterolemia (3.8% vs. 8.7%, p<0.001), and multivessel disease (96.8% vs. 73.9%, p<0.001). Fasting plasma glucose (6.54±3.77mmol/L vs. 5.88±2.25mmol/L, p=0.016), total cholesterol (6.66±3.05 mmol/L vs. 4.57±2.96 mmol/L, p<0.001), LDL cholesterol (2.63±0.59 mmol/L vs. 2.47±0.86 mmol/L, p=0.047), apoprotein A (Apo A) (0.99±0.10 mmol/L vs. 1.11±0.54 mmol/L, p=0.017) and serum creatinine (112.05±40.90 umol/L vs. 102.78±37.79 umol/L, p=0.019) were higher in low fT3 group. We also found that low fT3 patients had lower left ventricular ejection fraction (54.67±9.81% vs. 58.66±8.07%, p<0.001) and higher level of pro-B-type natriuretic peptide (NT-proBNP) than normal fT3 patients (2130.11±2247.99 pg/ml vs. 847.72±1533.63 pg/ml, p<0.001), indicating the impaired function of heart. TSH in low fT3 group was higher than normal fT3 group (1.76±1.51mU/L vs. 1.34±0.67mU/L, p=0.003). There were no significant differences in age, blood pressure, high sensitive C reactive protein (hsCRP), history of hypertension and smoking between the two groups.

**Table 1:** Baseline characteristics according to baseline fT3 level

Characteristics	Normal fT3	Low fT3	*P*-value
	n=402	n=126	
Female n (%)	99 (24.6)	45 (35.7)	0.023
Age (years)	68.9±10.1	70.2±10.8	0.181
SBP (mmHg)	132.49±21.77	130.06±14.30	0.234
DBP (mmHg)	75.30±12.46	76.52±11.77	0.323
Prior PCIn (%)	159 (39.5)	30 (23.8)	0.001
Diabetesn (%)	121 (30.1)	47 (37.3)	0.043
Hypertension n (%)	323 (80.3)	100 (79.4)	0.601
Hypercholesterolemia n (%)	35 (8.7)	48 (3.8)	<0.001
Cigarette smoking n (%)	122 (30.3)	36 (28.6)	0.738
Left ventricular ejection fraction (%)	58.66±8.07	54.67±9.81	<0.001
No. of diseased vessels n (%)			
1	104 (25.9)	4 (3.2)	<0.001
2	121 (30.1)	27(21.4)	<0.001
3	176 (43.8)	95 (75.4)	<0.001
In-hospital laboratory test			
Total cholesterol (mmol/L)	4.57±2.96	6.66±3.05	<0.001
Triglycerides (mmol/L)	1.63±0.98	1.59±0.89	0.715
HDL cholesterol (mmol/L)	1.03±0.27	0.98±0.16	0.043
LDL cholesterol (mmol/L)	2.47±0.86	2.63±0.59	0.047
ApoA1 (mmol/L)	1.11±0.54	0.99±0.10	0.017
ApoB (mmol/L)	0.85±0.41	0.79±0.16	0.104
Fasting plasma glucose (mmol/L)	5.88±2.25	6.54±3.77	0.016
hsCRP (mmol/L)	9.30±7.69	10.14±7.85	0.516
NT-proBNP (pg/mL)	847.72±1533.63	2130.11±2247.99	<0.001
Serum creatinine (umol/L)	102.78±37.79	112.05±40.90	0.019
fT4 (ng/L)	9.76±1.03	8.43±0.96	0.188
TSH (mU/L)	1.34±0.67	1.76±1.51	0.003

SBP: systole blood pressure, DBP: diastolic blood pressure, PCI: percutaneous coronary intervention, hsCRP: high sensitive C reactive protein, NT-proBNP: N-terminal pro-brain natriuretic peptide, fT3: free T3, fT4: free T4, TSH: thyroid stimulating hormone.

### Correlation of thyroid hormones with NT-proBNP and hsCRP

lnNT-proBNP was inversely related to fT3 (r=-0.443, p<0.001), but was not correlated with fT4 (r=0.052, p=0.24) and TSH (r=0.071, p=0.111). No significant correlations of lnhsCRP with fT3 (r=-0.068, p=0.125), fT4 (r=0.047, p=0.288) and TSH (r=0.055, p=0.209) were found.

### Outcomes with respect to health-related quality of life

**Figure 1:**
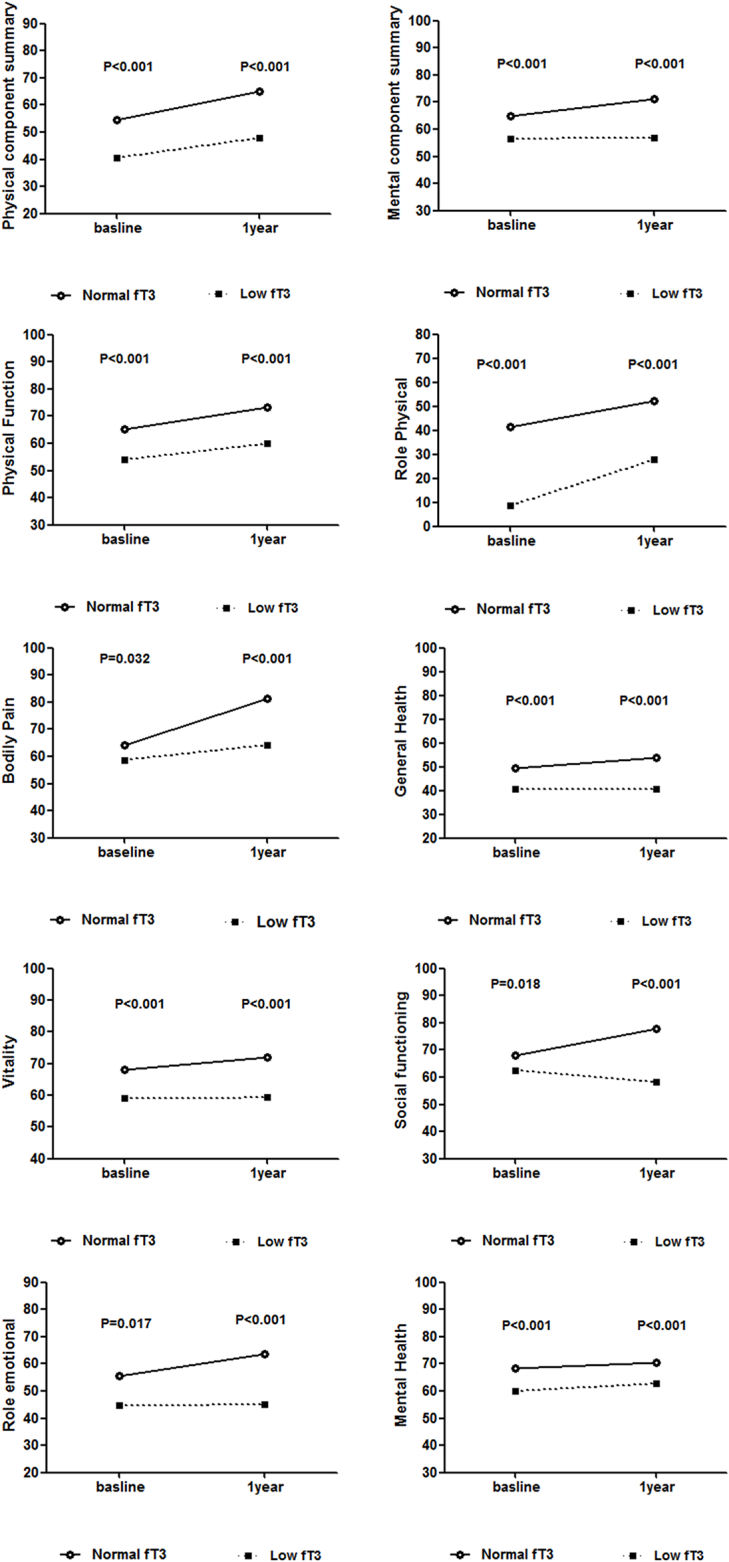
Unadjusted scores of HRQOL at baseline and 1-year following PCI according to baseline fT3 level

The unadjusted mean scores for each SF-36 domain are shown in Table 2 and Figure 1. For the overall population, HRQOL scores at 1-year follow-up were significantly higher than baseline for all dimensions of the SF-36 (all p<0.05), with improvements ranging from 3.7 points for mental health to 10.8 for physical health. (data not shown in Tables). Patients with low fT3 had lower scores for all domains of SF-36 both at baseline and 1-year follow-up compared with normal fT3 patients (all p<0.05). The Cronbach’s α coefficient of internal consistency was used to estimate the reliability of SF-36 domains. In all cases, Cronbach’s α was 0.83 for Physical Component Score (PCS) and was 0.87 for Mental Component Score (MCS). The value exceeded the minimum standard of 0.70. The minimal clinically important differences (MCIDs) for PCS and MCS were 8.18 and 6.02 respectively.

**Table 2:** Unadjusted scores of HRQOL at baseline and 1-year following PCI according to baseline fT3 level

SF-36 Subscale	Normal fT3	Low fT3	*P*-value
	n=402	n=126	
Baseline			
PCS	54.66±21.59	41.59±11.97	<0.001
Physical functioning	64.34±19.22	53.97±19.61	<0.001
Role physical	41.48±43.55	16.73±21.52	<0.001
Bodily pain	64.28±26.95	58.73±23.14	0.032
General health	49.55±17.86	40.63±13.49	<0.001
MCS	64.99±21.23	55.49±15.69	<0.001
Vitality	68.04±18.63	58.97±15.68	<0.001
Social functioning	67.07±23.99	62.52±18.85	0.018
Role emotional	55.64±44.83	44.71±45.05	0.017
Mental health	68.22±15.52	59.94±14.36	<0.001
1-year			
PCS	67.89±19.04	46.91±17.40	<0.001
Physical functioning	73.17±11.74	58.92±16.38	<0.001
Role physical	53.11±42.58	27.78±39.90	<0.001
Bodily pain	81.19±18.89	64.19±24.87	<0.001
General health	53.88±20.41	40.71±13.39	<0.001
MCS	71.34±17.65	56.86±19.54	<0.001
Vitality	72.04±17.29	59.30±15.98	<0.001
Social functioning	77.79±18.28	58.21±21.09	<0.001
Role emotional	63.76±43.21	44.97±44.29	<0.001
Mental health	70.37±16.06	62.73±20.21	<0.001

PCS: physical component summary, MCS: mental component summary.

**Figure 2:**
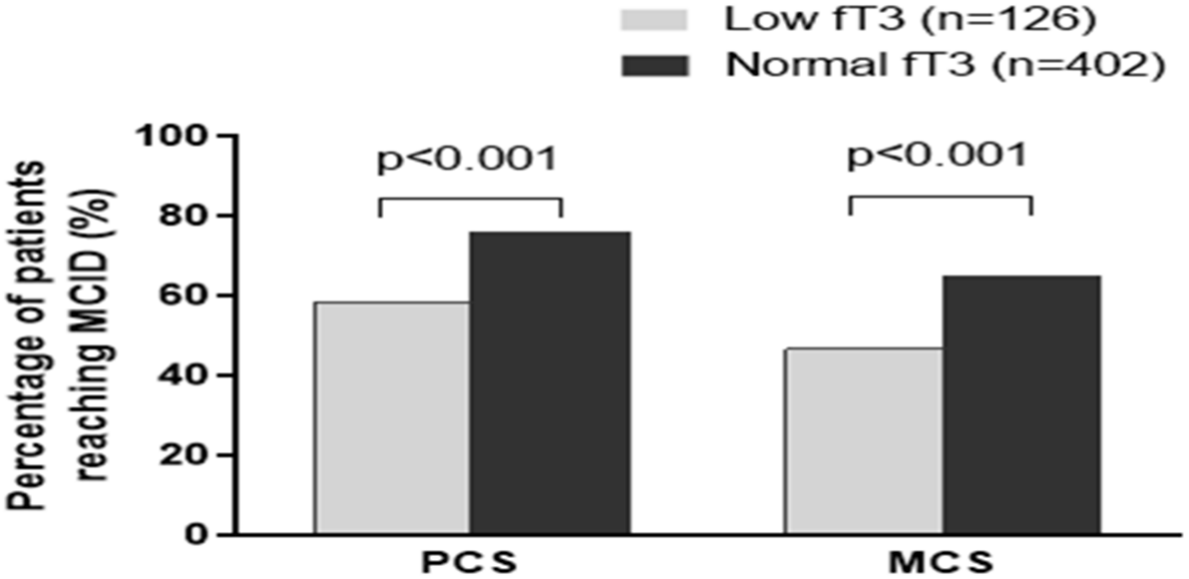
Percentage of patients reaching minimal clinically important difference (MCID) for Physical Component Score (PCS) and Mental Component Score (MCS) in patients with low or normal fT3 level.

After risk adjustment, low fT3 patients improved to a lesser extent than normal fT3 patients for 6 dimensions of health, including physical functioning (4.85±9.67 vs. 9.84±13.99, p=0.012), bodily pain (5.46±30.23 vs. 18.91±27.35, p<0.001), general health (0.79±12.5 vs. 5.33±17.705, p=0.010), vitality (0.34±16.57 vs. 4.00±15.24, p=0.022), social functioning (-3.21±17.31 vs. 9.82±22.19, p<0.001) and role emotional (1.26±49.90 vs. 8.13±51.10, p<0.001) independent of demographic characteristics (age, sex), comorbid medical conditions (hypertension, diabetes, hypercholesterolemia, prior PCI, smoking), other clinical factors (number of disease vessels, ejection fraction) (showed in Table 3). Generally, low fT3 patients demonstrated less improvement in PCS (6.03±14.61 vs. 12.22±19.84, p=0.008) and MCS (0.37±16.40 vs. 6.35±18.68, p=0.001) compared with normal fT3 patients. The improvements of quality of life for PCS and MCS in normal fT3 patients achieved MCID. Low fT3 group had lower percentage of patients reaching MCID for both PCS (58.7% vs. 76.1%, p<0.001) and MCS (46.8% vs. 65.2%, p<0.001) than normal fT3 group (Figure 2).

**Table 3:** Adjusted changes in HRQOL according to baseline fT3 level

Score change	Normal fT3	Low fT3	*P*-value
	n=402	n=126	
PCS	12.22±19.84	6.03±14.61	0.008
Physical functioning	9.84±13.99	4.85±9.67	0.012
Role physical	14.04±38.00	10.63±52.21	0.095
Bodily pain	18.91±27.35	5.46±30.23	<0.001
General health	5.33±17.70	0.79±12.5	0.010
MCS	6.35±18.68	0.37±16.40	0.001
Vitality	4.00±15.24	0.34±16.57	0.022
Social functioning	9.82±22.19	-3.21±17.31	<0.001
Role emotional	8.13±51.10	1.26±49.90	<0.001
Mental health	2.15±14.43	2.79±17.20	0.677

PCS: physical component summary, MCS: mental component summary.

Multivariate linear regression analyses showed that female (β=-3.98, 95%CI -7.76 to -0.16, p=0.041), cigarette smoking (β=-8.66, 95%CI -13.05 to -4.27, p<0.001), diabetes (β=-8.81, 95%CI -13.07 to -4.55, p<0.001), high level of NT-proBNP (β=-4.02, 95%CI -8.19 to -0.026, p=0.042) and low level of fT3 (β=0.22, 95%CI 0.062 to 0.382, p=0.007) were the independent risk factors for PCS improvement, and cigarette smoking (β=-4.70, 95%CI -9.26 to -0.14, p=0.043), diabetes (β-4.24, 95%CI -8.24 to -0.24, p=0.038), prior PCI (β=-5.94, 95%CI -8.35 to -3.37, p<0.001) and low level of fT3 (β=2.08, 95%CI 1.02 to 3.13, p<0.001) were the independent risk factors for MCS improvement (Table 4).

**Table 4:** Multivariate linear regression analysis for HRQOL changes at 1 year

Variables	PCS			MCS		
	β	95%CI	*P* value	β	95%CI	*P* value
Female	-3.98	-7.76 ∼ -0.16	0.041	1.21	-2.73 ∼ 4.91	0.621
Cigarette smoking	-8.66	-13.05 ∼ -4.27	<0.001	-4.70	-9.26 ∼ -0.14	0.043
Diabetes	-8.81	-13.07 ∼ -4.55	<0.001	-4.24	-8.24 ∼ -0.24	0.038
Prior PCI	-1.87	-5.65 ∼ 1.83	0.336	-5.94	-8.35 ∼ -3.37	<0.001
NT-proBNP	-4.02	-8.19 ∼ -0.026	0.042	-2.872	-7.147∼ -1.403	0.283
fT3	0.22	0.062 ∼ 0.382	0.007	2.08	1.02∼3.13	<0.001
Propensity Score	-0.053	-0.103 ∼ 0.018	0.101	-0.042	-0.087 ∼ 0.012	0.122

PCS: physical component summary, MCS: mental component summary, PCI: percutaneous coronary intervention, NT-proBNP: N-terminal pro-brain natriuretic peptide, fT3: free T3.

## DISCUSSION

The most remarkable result of our study was that we found a significant different HRQOL in ACS patients with different level of fT3. ACS patients with low fT3 had a diminished HRQOL than normal fT3 patients no matter at the first time of admission to our hospital or 1 year after DES implantation. Meanwhile, our intensive analyses showed that low fT3 patients had less improvements in physical and mental health after 1 year follow-up independent of demographic characteristics (age, sex), smoking) and other clinical factors (number of disease vessels, ejection fraction). Compared to normal fT3 patients, low fT3 patients had less likelihood to achieve MCID. Multivariate linear regression analyses also revealed that low level of fT3 was a predictor of worse PCS and MCS improvements after DES implantation. The improvements in most dimensions of SF-36 following 1 year after DES implantation were consistent with other studies [13-15].

Low fT3 syndrome is characterized by low fT3 levels with normal T4 and TSH, which is often accompany with chronic or severe disease. Previous studies have explored the incidence rate of low fT3 in ACS patients. Zhang et al. [12] enrolled a total of 501 patients with acute myocardial infarctions and found that 34.1% people had a low level of fT3. In a prospective, observational, and cross section study enrolled 400 ACS patients, Abdulaziz [16] found that low T3 syndrome was noticed in 10.2% of critically ill patients. Pfister et al. [17] assessed 615 consecutive patients hospitalized for cardiovascular disease and found 7.1% patients had low-T3 syndrome. Our study enrolled more than 500 ACS patients and found about 23.9% patients had a low fT3 level, indicating that low fT3 is a relatively large proportion in patients with coronary artery disease.

Thyroid hormone has an essential role in cardiovascular homeostasis. Clinical studies have revealed that low T3 syndrome had been linked to adverse cardiovascular prognosis in patients with ACS. Two studies by Özcan et al. [18] and Lazzeri et al. [19] involving consecutive ST-segment elevation myocardial infarction (STEMI) patients undergoing primary percutaneous coronary intervention revealed that the low T3 syndrome increased short- and long-term mortality. Brozaitiene et al. [20] conducted a study with patients attending a cardiac rehabilitation program after ACS and reported an association of lower fT3 levels with all-cause mortality. A recent study by Yazıcı et al. [21] also found that low T3 is related to increased early and late mortality in non ST-segment elevation myocardial infarction (NSTEMI) patients. As ACS mortality has decreased with improved therapies, the patient’s health status has been increasingly recognized as a critical outcome. Studies have found an association between cardiovascular risk factors such as smoking or diabetic and poor physical function [22], but rare studies explore the relationship of thyroid function especially low fT3 syndrome and HRQOL. A cross-sectional and small sample study with 122 coronary artery disease (CAD) patients enrolled by Bunevicius et al. demonstrated that reduced thyroid hormone availability was associated with poor physical and mental components of the HRQOL [23]. To our knowledge, this is the first study to examine the impact of low T3 syndrome on HRQOL in ACS patients who have undergone DES implantation. We choose the SF-36 to evaluate health state as it has been the most widely used generic measure of HRQOL in medical research including CAD [24, 25].

Previous studies have explored the effects of thyroid dysfunction including overt and subclinical thyroid diseases on the mental and physical health [26]. Studies have proved that overt thyroid failure was associated with impaired HRQOL [27-29]. The relationship between subclinical thyroid disease and HRQOL remains controversial. Bell RJ et al. [30] enrolled a total of 1423 non-healthcare-seeking women and found that subclinical thyroid disease in women was not associated with lower well-being or impaired health-related quality of life. While Chueire et al. [31] found that subclinical thyroid disease increased the risk for depression in the elderly. Up to now, studies about the influence of low fT3 syndrome on HRQOL especially in CAD were lacking. Our study has excluded patients with primary thyroid disease to avoid endocrine bias, and we found that low fT3 was associated with worse quality of life in ACS patients. At 1 year follow-up, low fT3 patients had less improvements of physical and mental health and less likelihood to achieve MCID compared to normal fT3 patients, suggesting that low fT3 is an independent predictor of worse level of quality of life in ACS patients. Our findings were consistent with some other studies on patients with other non-thyroidal somatic disorders. Bunevicius et al. revealed that lower free T3 concentrations were associated with shorter survival and worse HRQOL across physical and mental health domains in primary brain tumor patients [32, 33]. The reduced T3 concentration is an independent predictor of greater disease severity of brain tumor patients without clinical thyroid disorders. They still found that in ischemic stroke patients, lower free T3 concentration was an independent predictor of poor functional and cognitive outcomes assessed by modified Rankin Scale (mRS) and Mini-Mental State Examination (MMSE) [34]. Our findings indicate that the fluctuations of the thyroid hormone concentrations in the absence of overt thyroid disease could impact the health-status in ACS.

The exact mechanisms of effects of low fT3 on HRQOL are likely to be complex. Thyroid hormones are known to play an essential role in many biological processes in every tissue and could represent a catabolic state which had weak muscle strength and a propensity to fatigue. Bunevicius et al. documented that lower fT3 concentrations were associated with increased physical fatigue and exertion fatigue in patients with CAD [35]. In addition, thyroid hormones are essential in the nervous system development of the human brain [36]. A lack of thyroid hormones during human brain development could lead to irreversible mental retardation and neurological deficits [37], thus lead to diminished mental quality of life [38]. The sensitivity of brain to fluctuation in the thyroid hormone concentration might also be responsible for depression in patients with CAD [39].

Our study has several limitations. First, our study is a single central trial and the sample size of the study is small. Multi-center clinical trials with large sample size are demanded. Second, as with any other clinical trial, we did not complete follow-up quality-of-life data for all of the eligible population. Many patients were excluded from the study on the basis of unfavorable anatomy for PCI or they refused to participate or physically incapable of responding. Third, our results showed only the first year of follow-up after PCI, long- term effects are currently unknown.

In conclusion, the incidence of low T3 syndrome is prevalent in ACS patients. Low fT3 level was an independent predictor of worse HRQOL improvements. Considering the association of lower fT3 concentrations with adverse clinical outcomes and worse quality of life, patients with ACS are recommended to be evaluated for low T3 syndrome. Further studies are needed to explore whether treatment of low T3 syndrome can improve the prognosis and HRQOL of ACS patients.

## MATERIALS AND METHODS

### Study population

Consecutive patients with a diagnosis of ACS (including unstable angina and myocardial infarction with or without ST-segment elevation) who were treated with DES in Shanghai Ninth people’s hospital were enrolled in this study. All patients received PCI under local anesthesia and received optimal medical therapy. Between May 2012 and March 2014, 702 ACS patients were admitted to our hospital. 12 patients died in the hospital, 19 patients did not implant drug-eluting stent because of disease itself. 21 patients were excluded because they refused to participate. 37 Patients with over hyperthyroidism or hypothyroidism, subclinical hyperthyroidism or hypothyroidism, thyroid hormone replacement therapy and aminodarone therapy were also excluded. Finally, a total of 613 patients who completed the baseline HRQOL instrument constituted the study. All Patients were classified into low fT3 group (fT3<2.5 ng/L) and normal fT3 group (2.5 ng/L <FT3<3.9 ng/L) according to the serum fT3 level.

The study protocol was approved by hospital Ethics Committee of the institution and written informed consent was obtained in all patients.

### Demographic and clinical data

Data on patient demographics (age and sex), clinical and angiographic features (Left ventricular ejection fraction, blood pressure, number of diseased vessels), cardiovascular risk factors (diabetes, hypertension, hypercholesterolemia, cigarette smoking), and history of prior PCI were obtained through patient interview and review of medical records.

### Biomarker measurement

All patients had a blood collection after an overnight fast during the first 24-48 hours of admission before PCI. The serum glucose and lipid profiles were measured with Enzymatic colorimetric (HITACHI7170A Analyser, Japan). NT-proBNP was measured with electro-chemiluminescent immunoassay kit (Roche Diagnostics, Mannheim, Germany). hsCRP was determined using a high-sensitivity ELISA kit (Biocheck Laboratories, Toledo, OH). fT3, free T4 (fT4) and TSH, were assessed by chemiluminescence (Automatic Chemiluminescence Immune Assay System ACS180 with related kits; Bayer, Germany). The reference values were as follows: fT3, 2.5–3.9 ng/L; fT4, 5.8–16.4 ng/L; and TSH, 0.34–5.6 mU/L.

### HRQOL evaluation

HRQOL was evaluated with the Short-Form 36 (SF-36) questionnaire [40] for each study subject always by the same trained interviewer who did not know the thyroid hormone levels both at the time of enrollment and at 1 year follow-up after DES implantation. The baseline questionnaires were completed in hospital at the time of the initial revascularization procedure, subsequent questionnaires were sent by mail or by outpatient clinic. For those who did not response to the mailed survey more than 2 weeks, we connected them by telephone. This general HRQOL instrument was chosen rather than more specific tools since it provides an assessment of subjects’ own perception of their quality of life as a function of their general state of health. SF-36 includes 36-item scales measuring the following 8 health domains: physical functioning, role physical, role emotional, social functioning, bodily pain, mental health, vitality, and general health. Summary scores are derived by collapsing the 8 subscales, each scale ranges from 0 to 100, with a higher score corresponding to a better HRQOL. The 8 specific domains of physical and emotional scores can be summarized into 2 main scores: PCS and MCS.

### Clinical follow-up

All patients received clinical follow-up at 1 year after discharge. Major adverse cardiac events (MACE) including death, myocardial infarction (MI), stroke or repeat revascularization were recorded through direct patient interview in a special outpatient clinic or by indirect conversation with patients or their relatives by phones.

### Statistical analysis

Statistical analysis was performed using the SPSS statistical package, version 19.0 (SPSS Inc, USA). Continuous variables were expressed as mean ± SD and compared with 2-tailed, unpaired student t test. Categorical variables were presented as counts and frequencies and compared with Chi square test or the Fisher’s exact test. The strength of association between two continuous variables was evaluated with Pearson correlation analysis or Spearman rank order correlation test, as appropriate. As NT-proBNP and hsCRP values were markedly skewed, they were natural log-transformed prior to analysis. The reliability and internal consistency of the SF-36 were assessed via Cronbach’s α and the Nunnally criterion of 0.7 [41]. The MCID was used to evaluate whether the changes of the SF-36 score would be clinically meaningful [42]. The MCID was defined as the smallest difference in a score that patients perceived as beneficial and that could mandate a change in their management [43]. We used a distribution-based method to calculate the MCID values for the HRQOL instruments and applied the following formula: 1-SM [a change of 1 standard error of the mean (SEM)] = SD × √ (1-α), where α is the Cronbach’s reliability coefficient [44]. A change of 1 SEM was empirically demonstrated to correspond to the MCID in a previous study using the SF-36, which indicates that the 1-SEM criterion can be applied as a proxy for a clinically meaningful change.

A propensity score of probability in low fT3 level was used to adjust for potential bias between these groups. This was accomplished by performing a multivariable logistic regression analysis using low fT3 level as the dependent variable and entering all demographics, physical examination findings, smoking statue and clinical accompanying disease that were likely to affect the probability of low fT3 level. Stepwise backward elimination was employed and the resultant independent predictors of low fT3 level were then used to calculate the probability of low fT3 level (propensity score). By introducing the propensity score into regression adjustment, the effect of low fT3 level was estimated by adjustment for the impact of background covariates. The bias in the background covariates between the two groups could be removed by adjustments made with the propensity score.

Multivariate linear regression models were created to identify whether the mean change of quality of life of low fT3 patients differed from normal fT3 patients. Each regression model adjusted for demographic characteristics (age, sex), comorbid medical conditions (hypertension, diabetes, hypercholesterolemia, prior PCI, smoking), and other clinical factors (number of disease vessels, ejection fraction). The propensity score was forced into all the models as covariate to balance the potential bias. Multiple imputation strategy was employed to account for missing scores which could potentially produce selection bias from survey non-responders. The results of sensitivity analysis using imputed data were similar to analytic cohort and were not presented in this paper. All tests of significance were two-tailed and a P<0.05 was considered significant.

## References

[1] Wang W, Thompson DR, Ski CF, Liu M (2014). Health-related quality of life and its associated factors in Chinese myocardial infarction patients. Eur J Prev Cardiol.

[2] Spertus JA, Jones P, McDonell M, Fan V, Fihn SD (2002). Health status predicts long-term outcome in outpatients with coronary disease. Circulation.

[3] Soto GE, Jones P, Weintraub WS, Krumholz HM, Spertus JA (2004). Prognostic value of health status in patients with heart failure after acute myocardial infarction. Circulation.

[4] Danzi S, Klein I (2014). Thyroid disease and the cardiovascular system. Endocrinol Metab Clin North Am.

[5] Chowdhury D, Ojamaa K, Parnell VA, McMahon C, Sison CP, Klein I (2001). A prospective randomized clinical study of thyroid hormone treatment after surgery for complex congenital heart disease. J Thorac Cardiovasc Surg.

[6] De Groot LJ (1999). Dangerous dogmas in medicine: the nonthyroidal illness syndrome. J Clin Endocrinol Metab.

[7] Ascheim DD, Hryniewicz K (2002). Thyroid hormone metabolism in patients with congestive heart failure: the low triiodothyronine state. Thyroid.

[8] Klemperer JD, Klein I, Gomez M, Helm RE, Ojamaa K, Thomas SJ, Isom OW, Krieger K (1995). Thyroid hormone treatment after coronary artery bypass surgery. N Engl J Med.

[9] Iervasi G, Pingitore A, Landi P, Raciti M, Ripoli A, Scarlattini M, L'Abbate A, Donato L (2003). Low-T3 syndrome: a strong prognostic predictor of death in patients with heart disease. Circulation.

[10] Pingitore A, Iervasi G, Barison A, Prontera C, Pratali L, Emdin M, Giannessi D, Neglia D (2006). Early activation of an altered thyroid hormone profile in asymptomatic or mildly symptomatic idiopathic left ventricular dysfunction. J Card Fail.

[11] Friberg L, Werner S, Eggertsen G, Ahnve S (2002). Rapid down-regulation of thyroid hormones in acute myocardial infarction: is it cardioprotective in patients with angina?. Arch Intern Med.

[12] Zhang B, Peng W, Wang C, Li W, Xu Y (2012). A low fT3 level as a prognostic marker in patients with acute myocardial infarctions. Intern Med.

[13] Spertus JA, Salisbury AC, Jones PG, Conaway DG, Thompson RC (2004). Predictors of quality-of-life benefit after percutaneous coronary intervention. Circulation.

[14] Kim J, Henderson RA, Pocock SJ, Clayton T, Sculpher MJ, Fox KA, RITA-3 Trial Investigators (2005). Health-related quality of life after interventional or conservative strategy in patients with unstable angina or non-ST-segment elevation myocardial infarction: one-year results of the third Randomized Intervention Trial of unstable Angina (RITA-3). J Am Coll Cardiol.

[15] Li R, Yan BP, Dong M, Zhang Q, Yip GW, Chan CP, Zhang M, Zhang Q, Sanderson JE, Yu CM (2012). Quality of life after percutaneous coronary intervention in the elderly with acute coronary syndrome. Int J Cardiol.

[16] Abdulaziz Qari F (2015). Thyroid hormone profile in patients with acute coronary syndrome. Iran Red Crescent Med J.

[17] Pfister R, Strack N, Wielckens K, Malchau G, Erdmann E, Schneider CA (2010). The relationship and prognostic impact of low-T3 syndrome and NT-pro-BNP in cardiovascular patients. Int J Cardiol.

[18] Özcan KS, Osmonov D, Toprak E, Güngör B, Tatlısu A, Ekmekçi A, Kaya A, Tayyareci G, Erdinler İ (2014). Sick euthyroid syndrome is associated with poor prognosis in patients with ST segment elevation myocardial infarction undergoing primary percutaneous intervention. Cardiol J.

[19] Lazzeri C, Sori A, Picariello C, Chiostri M, Gensini GF, Valente S (2012). Nonthyroidal illness syndrome in ST-elevation myocardial infarction treated with mechanical revascularization. Int J Cardio.

[20] Brozaitiene J, Mickuviene N, Podlipskyte A, Burkauskas J, Bunevicius R (2016). Relationship and prognostic importance of thyroid hormone and N-terminal pro-B-type natriuretic peptide for patients after acute coronary syndromes: a longitudinal observational study. BMC Cardiovasc Disord.

[21] Yazıcı S, Kırış T, Ceylan US, Terzi S, Erdem A, Atasoy I, Emre A, Yeşilçimen K (2017). Relation of low T3 to one-year mortality in non-ST-elevation acute coronary syndrome patients. J Clin Lab Anal.

[22] Xue C, Bian L, Xie YS, Yin ZF, Xu ZJ, Chen QZ, Zhang HL, Wang CQ (2017). Impact of smoking on health-related quality of life after percutaneous coronary intervention treated with drug-eluting stents: a longitudinal observational study. Health Qual Life Outcomes.

[23] Bunevicius R, Staniute M, Gintauskiene V, Buneviciute J, Nemeroff CB, Brozaitiene J (2013). Endocrine associations with health-related quality of life in coronary artery disease patients. Int J Cardiol.

[24] Krumholz HM, Cohen DJ, Williams C, Baim DS, Brinker J, Cabin HS, Heuser R, Hirshfeld J, Leon MB, Moses J, Savage MP, Cleman M (1997). Health after coronary stenting or balloon angioplasty: results from the Stent Restenosis Study. Am Heart J.

[25] Pocock SJ, Henderson RA, Seed P, Treasure T, Hampton JR (1996). Quality of life, employment status and anginal symptoms after coronary angioplasty or bypass surgery. Circulation.

[26] Gulseren S, Gulseren L, Hekimsoy Z, Cetinay P, Ozen C, Tokatlioglu B (2006). Depression, anxiety, health-related quality of life, and disability in patients with overt and subclinical thyroid dysfunction. Arch Med Res.

[27] Bianchi GP, Zaccheroni V, Solaroli E, Vescini F, Cerutti R, Zoli M, Marchesini G (2004). Health-related quality of life in patients with thyroid disorders. Qual Life Res.

[28] McMillan CV, Bradley C, Woodcock A, Razvi S, Weaver JU (2004). Design of new questionnaires to measure quality of life and treatment satisfaction in hypothyroidism. Thyroid.

[29] McMillan C, Bradley C, Razvi S, Weaver J (2008). Evaluation of new measures of the impact of hypothyroidism on quality of life and symptoms: the ThyDQoL and ThySRQ. Value Health.

[30] Bell RJ, Rivera-Woll L, Davison SL, Topliss DJ, Donath S, Davis SR (2007). Well-being, health-related quality of life and cardiovascular disease risk profile in women with subclinical thyroid disease-a community-based study. Clin Endocrinol (Oxf).

[31] Chueire VB, Romaldini JH, Ward LS (2007). Subclinical hypothyroidism increases the risk for depression in the elderly. Arch Gerontol Geriatr.

[32] Bunevicius A, Laws ER, Deltuva V, Tamasauskas A (2017). Association of thyroid hormone concentrations with quality of life of primary brain tumor patients: a pilot study. J Neurooncol.

[33] Bunevicius A, Deltuva VP, Tamasauskas S, Smith T, Laws ER, Bunevicius R, Iervasi G, Tamasauskas A (2017). Preoperative low tri-iodothyronine concentration is associated with worse health status and shorter five year survival of primary brain tumor patients. Oncotarget.

[34] Bunevicius A, Kazlauskas H, Raskauskiene N, Janusonis V, Bunevicius R (2014). Ischemic stroke functional outcomes are independently associated with C-reactive protein concentrations and cognitive outcomes with triiodothyronine concentrations: a pilot study. Endocrine.

[35] Bunevicius A, Gintauskiene V, Podlipskyte A, Zaliunas R, Brozaitiene J, Prange AJ, Bunevicius R (2012). Fatigue in patients with coronary artery disease: association with thyroid axis hormones and cortisol. Psychosom Med.

[36] Bernal J (2007). Thyroid hormone receptors in brain development and function. Nat Clin Pract Endocrinol Metab.

[37] Fierro-Benitez R, Ramirez I, Garces J, Jaramillo C, Moncayo F, Stanbury JB (1974). The clinical pattern of cretinism as seen in highland Ecuador. Am J Clin Nutr.

[38] Shin YW, Choi YM, Kim HS, Kim DJ, Jo HJ, O'Donnell BF, Jang EK, Kim TY, Shong YK, Hong JP, Kim WB (2016). Diminished quality of life and increased brain functional connectivity in patients with hypothyroidism after total thyroidectomy. Thyroid.

[39] Bunevicius R, Varoneckas G, Prange AJ, Hinderliter AL, Gintauskiene V, Girdler SS (2006). Depression and thyroid axis function in coronary artery disease: impact of cardiac impairment and gender. Clin Cardiol.

[40] Ware JE, Sherbourne CD (1992). The MOS36-item short-form health survey (SF-36).I. Conceptual framework and item selection. Med Care.

[41] Nunnally JC, Bernstein IH (1994). Psychometric Theory.

[42] Carlson ML, Tveiten ØV, Yost KJ, Lohse CM, Lund-Johansen M, Link MJ (2015). The minimal clinically important difference in vestibular schwannoma quality-of-life assessment: an important step beyond P <.05. Otolaryngol Head Neck Surg.

[43] Brozek JL, Guyatt GH, Schunemann HJ (2006). How a well-grounded minimal important difference can enhance transparency of labelling claims and improve interpretation of a patient reported outcome measure. Health Qual Life Outcomes.

[44] Wells G, Beaton D, Shea B, Boers M, Simon L, Strand V, Brooks P, Tugwell P (2001). Minimal clinically important differences: review of methods. J Rheumatol.

